# A qualitative approach to identify barriers to multi-professional teamwork among medical professors at Iranian teaching hospitals

**DOI:** 10.1186/s12913-021-06421-4

**Published:** 2021-05-20

**Authors:** Hakimeh Hazrati, Seyed Kamran Soltani Arabshahi, Shoaleh Bigdeli, Mozhgan Behshid, Zohreh Sohrabi

**Affiliations:** 1 Center for Educational Research in Medical Sciences (CERMS), Department of Medical Education, School of Medicine, Iran University of Medical Sciences, Tehran, Iran; 2 Medical Education Research Center, Health Management and Safety Promotion Research Institute, Tabriz University of Medical Sciences, Tabriz, Iran

**Keywords:** Medical education, Healthcare, Teamwork, Teaching hospitals, Multi-professional, Qualitative research

## Abstract

**Background:**

In some cases of diseases, such as infectious, neurological and chronic ones prevention and treatment is complex. Therefore, a single medical specialty alone cannot effectively manage treatment of patients due to health care needs of them and complexities of treatment. Instead, a team composed of different healthcare disciplines with effective, continuous, and organized communication must follow up various aspects of patient care. In this regard, the present qualitative study aimed to shed light on the experiences of clinical teachers of multi-professional teamwork barriers within Iranian teaching hospitals.

**Methods:**

In this qualitative research, the experiences of medical clinical teachers of multi-professional teamwork barriers within teaching hospitals were explained. Sampling was theoretical and the data were collected from experienced clinical teachers and medical students studying at several Universities of Medical Sciences through semi-structured interviews and observation, which were continued until data saturation. Fifteen clinical teachers and five medical students participated in the study. The interviews were analyzed using conventional content analysis.

**Results:**

Three main categories were extracted. The first category was enhancing the culture of interdisciplinary education included paving the way for an interdisciplinary culture, enhancing teamwork culture, and having a general view of medical sciences instead of specialization. The second category was barriers of interdisciplinary education included influence of the dominant culture of specialization in society, poor interdisciplinary education infrastructure, and individualism as a value of society. And the third category was consequences of specialization included medical sciences education under the shadow of specialization, possibility to harming patients, and distrust of society in the services provided by the 1st and 2nd level centers.

**Conclusion:**

It seems that attitudinal barriers, teamwork difficulties, and the culture of individualism are evident in Iran; more, roles of the healthcare team and the status of each member is not clear. Designing interactive curriculum and arranging clinical settings to facilitate exchange of ideas among clinical teachers and students of different disciplines, is a step forward to achieving a common value concept, language, and common perception, and establishing cooperation and understanding among disciplines involved, which leads to further understanding of the professional responsibilities of other disciplines.

**Supplementary Information:**

The online version contains supplementary material available at 10.1186/s12913-021-06421-4.

## Background

In some cases of diseases such as infectious, neurological and chronic ones, prevention and treatment is complex [1, 2]. Therefore, the treatment process cannot be managed effectively via a single medical specialty because patients have diverse healthcare needs and disease prevention and treatment becomes complex [3]. Appropriate health care delivery damages from poor or nonexistent cooperation between team members, failure to share relevant information between health professionals, and poor interpersonal skills [4]. Instead, a team composed of different healthcare disciplines related to effective, continuous, and organized communication must follow up on various aspects of providing health care [5, 6]. Safe and effective care is dependent not only on the knowledge, skills and behaviors of front-line workers, but also on how those workers cooperate and communicate in the work environment, which itself is usually part of a larger organization. In other words, patients are dependent to the health care system and health care workers who do the right thing at the right time [4]. According to the American Medical Association, patients receive high-quality and safe care when healthcare professionals effectively work as a team with constructive communication and mutual understanding, respect, and trust [7].

Due to the nature of providing healthcare as a team, greater focus has been given to the development of communication skills among healthcare disciplines and interdisciplinary education has received considerable attention [3]. In the Gawande et al. study, conducted in three Massachusetts teaching hospitals located in Boston city in the United States of America, on investigation of incidence and types of adverse events and negligent care in surgery, it is indicated that from 146 surgery incidents, 43% were preventable medical errors resulting from poor communication between different professions [8]. Interdisciplinary education is defined by the World Health Organization (WHO) as a method by which a group of students of various healthcare professions learn from one another in a specified period and location with the aim of establishing interaction and cooperation, in order to offer healthcare, prevention, treatment, and rehabilitation services and health promotion [9]. In Iran and most developing countries, students of medical sciences are still taught in a mono-disciplinary manner [10]. The students receive instructions in separate classes or in clinical educational settings, with no opportunity for interaction or sharing information, recognizing different roles, understanding differences, similarities, and capabilities, or a common responsibility and goal to provide a basis for team care provision [11].

In the current medical education and healthcare settings, there is an increase in preventable medical errors, mortality, complications of disruptive and disparate treatments, inconsistency in multilateral treatment processes, contradictory and parallel treatments, nosocomial infections, and thus prolonged hospital stay and increased costs [12]. The provision of responsibility-based services to patients instead of meeting patients needs has led to dissatisfaction of service providers and clients [13]. These indicate the failure of the medical education system (specialist and single-discipline education) in meeting the needs and overcoming the challenges of the healthcare system, further clarifying the need for revolutionizing the medical education system and the integration of medical education [14]. For improving health care, we need to shift the focus from individualism to a system approach and look at health care as a whole system, with all its complexities and interdependence. We should focus more on the transparency of the processes of care rather than focusing solely on the single act of care [4] .The implementation of interdisciplinary education requires evaluating cultures, beliefs, and values governing current educational practice. Quantitative studies have assessed attitudes of different groups of students and clinical teachers in Iran. The attitudes of students and professors with regard to interdisciplinary education were moderate, and the attitude of medical students were moderate to low [15]. In addition, attitudinal differences between the disciplines of health sciences are the most important disturbing factors in interdisciplinary education, while some medical students even regarded this type of education as a waste of time [15,17]. It seems that the qualitative approach is the best method to find the root of difference in attitudes towards interdisciplinary education and its barriers in developing societies such as Iran. Thus, this qualitative study aimed to identify barriers to multi-professional teamwork among medical professors at Iranian teaching hospitals. The findings can offer insights to planners for institutionalizing the culture of interdisciplinary education and changing the curriculum of medical disciplines based on interdisciplinary education, appropriate for the Iranian context.

## Methods

### Study design

**Fig. 1:**
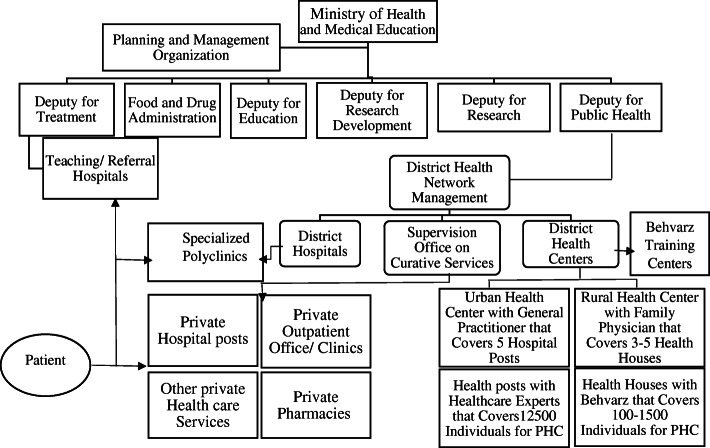
The structure of the health care system in Islamic Republic of Iran [18]

This qualitative study used face-to-face semi-structured interview to explore the lived experiences of clinical teachers to identify barriers to multi-professional teamwork among medical professors at Iranian teaching hospitals. The health care system in Islamic Republic of Iran is established in three levels of district, province and the country. District level is the smallest independent unit in the health system of the country. Its executive units include Health House (Health post), Health Base, Urban Health Center, Rural Health Center, Behvarz Training Center, District Health Center, District Hospital, and District Health Network Management [18]. The network of rural health houses is supported by rural health centers which are staffed by technicians and administrative personnel working under the supervision of a physician. In urban areas, the urban health centers provide ambulatory care. This network of urban and rural PHC facilities is supported by district hospitals. Located in cities, these general hospitals offer a variety of specialist services. In large cities, which often act as provincial capitals, provincial hospitals affiliated to the Ministry of Health and Medical Education (MOHME), Ministry of Welfare and Social Security and the private sector provide secondary and tertiary care [18]. Figure1 shows the current structure of the health system in Iran. We conducted 15 key informant interviews with clinical teachers and 5 medical students. A total of 20 semi-structured interviews conducted between November 2019 and May2020. The original interview guide including open-ended questions were piloted with mangers of 3 education development centers. Four teaching hospitals in the Iran University of Medical Sciences were selected for taking field notes.

#### Participants and sampling

Theoretical sampling was conducted, and data were collected from experienced clinical teachers who were willing to share their experiences. Some of whom had Education Development Center (EDC) management experience. Interviews were conducted with clinical teachers of Iran, Shahid Beheshti, Tabriz, Isfahan, Shiraz, Mashhad, Mazandaran, and Guilan Universities of Medical Sciences. To develop and complete the categories, medical students who were willing to participate were interviewed as well. Data collection continued until data saturation, when no new piece of data was emerged.

### Data collection and analysis

Semi-structured interviews were used as the main data collection technique. Observation and field note taking were also used. With permission of managers of the teaching hospitals (Rasoul Akram Tehran, Firoozgar Tehran, Imam Reza Tabriz, Shahid Madani Tabriz), one of the researchers (HH) visited each hospital and took notes of important points observed in the real contexts of journal club sessions, morning reports and educational rounds. The interviews were conducted at a quiet place and at a time convenient for the participants. In the interview sessions, after explaining the study goals and methods as well as securing informed consent, the interviews were recorded with a voice recorder, and notes on important points were taken after obtaining the participants permission. The interviews has been commenced with open guided questions in order to encourage the participants to speak freely and express their personal experiences about clinical teaching; for example, Can you talk about one of your best interdisciplinary teaching experiences with medical students in an outpatient setting or patient bedside? What were the characteristics of that? As the interview progresses, the probing questions become more specific, allowing a deeper exploration of the questions. At the end of each interview, the content was transcribed verbatim. Two initial interviews were piloted to improve interview questions. The interviews were listened to and the transcripts were read and re-read by two members of the research team for several times, so that they could be immersed in the text. Semantic units were specified by highlighting sentences in each paragraph and the open coding (first level) process commenced by emphasizing the explicit and implicit content, and each unit of analysis received a code. Subsequently, the codes were classified into sub-categories and categories, based on their spectrum and attributes. As data analysis continued, the codes were repeatedly reviewed, and contradictions were resolved by discussion. At the end, a free discussion was conducted with the research team who systematically checked and re-checked the categories extracted from the interviews and reviewed the emerging ideas in the categories and made logical connections between categories and subcategories. Theoretical sampling was conducted on the basis of theoretical ideas and concepts revealed in the process analysis to expand, refine and complete the categories.

### Rigor of the study

To maintain trustworthiness of the study, four criteria of credibility, confirmability, dependability, and transferability were taken into account [19]. For credibility, the interviewer tried to attract participants trust by making a good rapport with them and the extracted codes has been sent to the interviewees for confirmation. For triangulation, interviews were held with instructors of different clinical fields and medical students from different educational levels. Methodology and extracted codes were checked with expert instructors in qualitative studies and experts in medical education. Also, by the long-term engagement with data, data immersion was fulfilled. And also, researchers considered bracketing during data collection and analysis, and bracketed their prior experiences with clinical teaching and clinical settings and their own beliefs about effective interdisciplinary education. For data confirmability, data collection and analysis, decision making for coding and classification, and other steps were documented. For dependability, the extracted codes were reviewed in the panel of researchers and were either confirmed or other codes were selected. To promote transferability of findings, an external researcher who was expert in the field of qualitative studies was asked to analyze the steps and data collection processes. One of the researchers (HH) was also asked to comment on the systematic implementation of study steps. For maximum variation sampling, the interviews with instructors of diverse universities, clinical fields, age ranges, genders, and positions were carried out.

## Results

Fifteen clinical teachers and five medical students participated in the interviews. Four participants were Educational Development Center (EDC) managers, three from the emergency medicine department, five from the internal medicine department, two from the surgery department and one from the urology department. Two educators were full professors, while 13 were associate professors. (Table1).

**Table 1:** Personal and social characteristics of the study participants

Group	Participant number	Academic degree/	Work experience	Age	Gender
Educational Development Center (EDC)	P4	Professor	21	48	Male
	P8	Professor	18	45	Female
	P11	Associate Professor	25	52	Male
	P13	Associate Professor	19	56	Male
Emergency medicine	P2	Associate Professor	15	40	Male
	P3	Associate Professor	16	45	Male
	P12	Associate Professor	18	42	Male
Internal medicine	P1	Associate Professor	25	58	Female
	P5	Associate Professor	27	53	Male
	P6	Associate Professor	26	54	Male
	P7	Associate Professor	23	47	Male
	P15	Associate Professor	16	49	Male
Medical student	P16	Intern, Urology Group		27	Male
	P17	Intern, Neurosurgery Group		24	Female
	P18	Intern, General surgery Group		26	Male
	P19	Intern, Gastroenterology Group		25	Male
	P20	Extern, ENT Group		24	Female
Surgery	P9	Associate Professor	28	56	Male
	P10	Associate Professor	28	53	Male
Urology	P14	Associate Professor	27	55	Male

Following data analysis, three main categories of enhancing the culture of interdisciplinary education, barriers of interdisciplinary education, and consequences of specialization were extracted.

In the enhancing the culture of interdisciplinary education category, three sub-categories were extracted paving the way for an interdisciplinary culture, enhancing teamwork culture, and having a general view of medical sciences instead of specialization (Table 2).

**Table 2:** Enhancing the culture of interdisciplinary education

Open code	Sub-Category	Category
Putting subspecialists together to initiate interdisciplinary interactions	Paving the way for an interdisciplinary culture	Enhancing the culture of interdisciplinary education
Merging subspecialty wards into two wards of internal medicine and surgery		
Placing the major responsibility on internists and surgeons, and merely consulting with subspecialists		
Necessitating the presence of internists among other specialists		
Consulting with other clinical teachers to resolve patients problems		
Examining the scenarios of morning reports from the view of clinical teachers of different specialties		
Holding meetings for complicated cases by involving clinical teachers of different wards		
Holding sessions with clinical teachers of relevant disciplines to discuss a patient case		
Holding continuous education programs for different specialties of medical sciences		
Defining inter-professional relationships in the curriculum of medicine		
Forming a healthcare team of specialists to perform different procedures if complications arise		
Having medical students learn from other disciplines such as nursing	Enhancing teamwork culture	
Establishing communication between nurses and medical students to answer the questions that arise		
Showing the importance of other disciplines in the process of patient care and the team nature of treatment		
Establishing communication between students and other disciplines in case of specialized questions		
Accepting the nature and role of other disciplines in diagnosis and treatment		
Establishing a horizontal relationship between general practitioners, specialists, and subspecialists		
Viewing different levels of medicine, from undergraduate to subspecialty, equally		
Delegating the main responsibility of treatment to an internist instead of a subspecialist	Having a general view of medical sciences instead of specialization	
Setting the educational atmosphere for interdisciplinary education		
Having internists manage the treatment because of their general view of patients for accurate diagnosis		
The subspecialists not commenting due to lack of a specialist view		
Subspecialists working alongside internists only to assist making diagnosis		

### Paving the way for an interdisciplinary culture

The first step towards implementation of interdisciplinary education is changing the norms and valuing interdisciplinary education. By offering interdisciplinary educational opportunities in different clinical education settings, the paradigm can be changed from specialization towards interdisciplinary education. A participant from the internal medicine department said: *An internist, pathologist, pharmacologist, and even a radiologist and surgeon can be present in morning reports, and each of them can explain their special scope. Students can learn from all of them and that is when extensive learning takes place.* (P5). Another participant from the same department mentioned: *Our counseling is provided on paper only. They write their suggestions down, but dont explain the reasons for their requests in person. Things we have in mind can occur much more effectively during face to face counseling sessions* (P6). An EDC manager declared: *We cannot implement such things in the regular curriculum. Wed better start from continuous education, because all specialists take part in the programs. They firmly opposed it at first; but then they said it was really good, and that they learned much from each other* (P8). A participant from the surgery department recounted: *We have a meeting with the basic sciences departments and academic staffs of anatomy department on Mondays. Neurosurgery is closely related to anatomy. Sometimes they discuss pure topics of anatomy, and we explain their applications in surgery* (P10). A participant from the emergency medicine department said: *Each educator must define a back-up for the procedures he/she performs, so that we know who to link if a complication arises* (P2). An intern said: *We had a professor in the cardiology ward who introduced us to different wards. We communicated with all these wards and followed up on patients. We understood the roles of other wards in the treatment process, and the patient follow-up was accelerated* (P16).

### Enhancing teamwork culture

Teamwork attitude towards treatment: Cultural challenges, such as individualism and the superiority of doctors have weakened the teamwork spirit in the healthcare team. In this regard, a participant from the emergency medicine department said: *Teamwork is learned in the field. If interns ask questions, I tell them to consult a first-, second- or third-year resident, and then report the result of the counseling to me. If interns suggest that surgery is required, I will tell them to involve the surgeon, too. In this way, students learn that its not just them, but the surgeon, the residents, the neurosurgeon, the attending doctor, and they are all involved in the process of diagnosis and treatment.* (P2). An EDC manager mentioned: *When an intern doesnt know how to perform injection, he/she can watch a nurse or supervisor who is injecting a patient. Whats wrong with that? Interns shouldnt dwell on the fact that theyre doctors. Disciplines must accept each other* (p11).

### Having a general view of medical sciences instead of specialization

Due to the nature of medicine, which deals with human beings, and the interrelationship between various organs in the human body, we need a general view of medicine and must break the barriers separating sub-disciplines. A participant from the emergency medicine department mentioned: *In the emergency department, we communicate with all specialties. We have a general view there, but need to consult other disciplines after the general path is determined for the patient* (P12). Another participant from the same department commented: *Specialization has lost its meaning in the medical world. Other countries have only three departments: internal medicine, surgery, and emergency medicine. Other specialties can offer counseling if it is needed* (P3). A participant from the surgery department mentioned: *We have a tunnel vision when it comes to diseases. The organs cannot be seen as separate from each other; therefore, you cannot separate specialties from one another* (P9).

In the barriers of interdisciplinary education category, three sub-categories were extracted: influence of the dominant culture of specialization in the society, poor interdisciplinary education infrastructure, and individualism as a value of the society (Table 3).

**Table 3:** Barriers of interdisciplinary education in Iranian teaching hospitals

Open code	Sub-category	Category
Defining subspecialty domains, poor communication between specialties, and isolation of specialties	Influence of the dominant culture of specialization in the society	**Barriers of interdisciplinary education**
Failing to recognize the role of other disciplines in a specialized or professional domain		
Lack of a vertical relationship between specialists and subspecialists		
The main goal of medical students being the selection of a specialty and focusing their studies on that specialty		
The society trusting subspecialists more than internists and general practitioners		
Failing to define interdisciplinary interactions in the curriculum	Poor interdisciplinary education infrastructure	
Unpreparedness of departments for interdisciplinary education		
Separation of departments		
Lack of communication between medical faculties		
Absence of new contents of professional knowledge for interdisciplinary education		
Lack of cultural context and acceptance of professors to incorporate interdisciplinary education		
Unfamiliarity of Medical teachers and students with the philosophy of interdisciplinary education		
Negative attitudes towards teamwork, and lack of cooperation among faculty members	Individualism as a value of the society	
Dominant spirit of individualism		
Medicine and other disciplines looking down on each other		
Unwillingness to recognize the role of other professions in the process of treatment and rehabilitation		

### Influence of the dominant culture of specialization in the society

Isolated sub-specialties lead to loss of a general view of medicine and disrespect for the human being as a whole in diagnosis and treatment. Communication among disciplines has declined, and every discipline has its own territory. They raise these walls every day, and do not let other disciplines enter their territory. As stated by a participant from the internal medicine department: *Every discipline has a territory in which no party is allowed to enter. If these barriers are removed and people understand status of others, these problems can be avoided* (P7). Another participant from the internal medicine department mentioned: *Its interesting that specialists look down on each other. Instead of examining a certain aspect of the disease and referring the client to each other, they believe the disease belongs to their specialty* (P1). A participant from the internal medicine department said: *From the first day, students are concerned with the easiest, cleanest, and most profitable specialty, and pursue it. They examine the other wards only to the extent that they get a pass score* (P6). Another participant from the internal medicine department said: *Specialties have built up walls around them. If you ask them something about another part of the body, they claim that it is not their specialty, as if they have not studied general medicine at all* (P6).

### Poor interdisciplinary education infrastructure

Implementation of interdisciplinary education requires large-scale planning and changing the curriculum of medical sciences disciplines, content production and empowerment of professors based on an interdisciplinary view. An EDC manager declared: *In the curriculum of medical sciences, the status of other disciplines has not been defined. I think we must start from internship programs. However, departments do not agree to this because its coordination is challenging* (P11). A participant from the internal medicine department mentioned: *Sessions between different departments are rarely held, and each department performs its own duties. Each department claims that certain courses belong to them and they should teach them. They do not care what other departments do* (P6). Another participant from the same department commented: The major problem is the content, w*ritten by each discipline for itself. Our policy-makers have not done anything for the development of interdisciplinary contents thus far. It is not just about students being together; but, clinical educators must also have the teamwork spirit and have specific content for this approach*. (P6).

### Individualism as a value of the society

In teaching hospitals, healthcare teams are weak and vulnerable. Communication between various disciplines may become more complicated with assumptions such as the status of different disciplines and superiority of some over the others. Specific forms of knowledge and power may find superiority, thus affecting the professional relationship between team members. A participant from the emergency medicine department declared, *These things must be taught in pre-school. We always say I and are not comfortable with We* (p2). Another participant from the same department said: *[We think] one discipline is superior to another. We look down on each other. There is one department that ranks first, and one specialty that is the most popular. Full professors find it beneath their dignity to participate in morning report sessions or transfer their experiences to an assistant professor...* (p3). A participant from urology department mentioned: *When specialists make a diagnosis and show off their skills, everythings over. Specialists only consider their own role. So, they do not care how the nurse behaves or whether the patient needs referral to rehabilitation, or to follow up on the results of the rehabilitation* (p14).

In the category of consequences of specialization, three subcategories were extracted: medical sciences education under the shadow of specialization, possibility to harming patients, and distrust of the society in the services provided by the 1st and 2nd level centers (Table 4).

**Table 4:** Consequences of specialization in the culture of Iran

Open code	Sub-category	Category
Resident-oriented education in clinical wards	Medical sciences education under the shadow of specialization	**Consequences of specialization in the culture of Iran**
Resident-oriented education and abandoning undergraduate medical education		
Residents and fellows responsibility for teaching the undergraduate medical students instead of professors		
Undergraduate medical students confusion as a result of complicated discussions in educational rounds		
Unrealistic self-confidence of undergraduate medical students in terms of having specialized skills		
Absence of teamwork between specialists, and thus harming the patients	Possibility to harming patients	
Isolated subspecialties imposing a financial burden on patients		
Specialization as a cause of not claiming responsibility for complicated cases		
Increasing medical errors		
Specialists not supporting one another if complication arises, which threatens the life of patients		
Busy wards, and all patients visiting specialized centers	Distrust of society in the services provided by the 1st and 2nd level centers	
Unfamiliarity of society with the status of specialized centers, and visiting 1st level centers without referral		
Busy specialized and subspecialized clinics since patients only visit specialists		
Trust in subspecialists and the internist, and general practitioners lack of status		

### Medical sciences education under the shadow of specialization

Education in teaching hospitals has become resident-centered, and subspecialty discussions are mostly held in educational rounds. The qualifications expected by the curriculum of undergraduate medical education have been neglected. A participant from the internal medicine department said: *Education has become resident-centered in the wards. In our wards, we simultaneously teach fellows, residents, interns, and externs. Most of the time, the discussions are so specialized that we feel the extern is distracted. There are many undergraduate medical students with the residents and fellows and they do not have any specifically defined responsibility. Most of our students do not have access to residents, and attending doctors expect that residents provide the necessary training. In fact, general medicine has been neglected* (P7). An extern student said: *Sometimes we just stand by and watch the subspecialty discussions; cases we may never ever encounter, because we may not choose that specialty* (p19).

### Possibility to harming patients

The ultimate goal of education and treatment is improving the quality of treatment. Poor interdisciplinary communication imposes the heaviest burden on patients. Patients have insufficient information about their disease and the process of treatment. Therefore, doctors must communicate with other disciplines, and seek consultation whenever deems necessary. A participant from the internal medicine department mentioned: *A patient may have a complication during endoscopy. The gastroenterologist says nothing to the patient, and the patient will need surgery because of this complication. The poor patient would probably not need surgery if he/she knew about the problem sooner; or if the gastroenterologist called a surgeon and consulted with him/her about the complication..*. (p15). A participant from the emergency medicine department said: *A patient admitted to the emergency department developed a complication as a result of ozone therapy. The neurosurgeon would not agree to operate on the patient* (p2). A participant from the internal medicine department mentioned: *Graduates who did not have the necessary skills due to inaccurate training felt they had the skills. Undergraduate medical students enter the subspecialty or specialty ward, and are assigned responsibilities they should not have. When the students graduate, they think they know all the processes or acquired the necessary skills in a ward just by passing 20 days there. You see such problems in society; patients complain, develop complications, and are faced with serious problems* (p13).

### Distrust of society in the services provided by the 1st and 2nd level centers

Iranian society deeply trusts specialists, and patients prefer to be visited by specialists directly and thus guaranteed an accurate diagnosis. This leads to overcrowded specialty and subspecialty clinics, loss of the general practitioners status, and undermining of their roles in the treatment of patients who require 1st level care. A participant from the emergency medicine department said: *The emergency department is overcrowded, partly because patients visit the emergency department even for a simple stomachache. They could simply go to the clinics in their neighborhood, and a general practitioner could treat them just the same. Unfortunately, people do not trust these clinics, and they want a subspecialist to diagnose their problem* (p3). An EDC manager declared: *Sometimes the society is not to be blamed; the services at 1*^*st*^ *level centers are not adequate, and patients lose their trust* (p8). A participant from the internal medicine department said: *Our subspecialty clinics are overcrowded, while general practitioners have nothing to do (p7). An intern student said: Nobody wants to remain a general practitioner, because no one takes them seriously. We try to choose a specialty as soon as our internship begins* (p20).

## Discussion

This qualitative study aimed to explain the experiences of clinical teachers in institutionalizing interdisciplinary education within Iranian teaching hospitals. Three main categories of enhancing the culture of interdisciplinary education, barriers of interdisciplinary education, and consequences of specialization were extracted. It seems that negative attitudes towards teamwork and the culture of individualism are deeply rooted in the culture of some societies [20]. While the status of the healthcare team members is not clear, and they are not familiar with each others role and significance of the patient care process, each team member performs one task separately, that resembles disconnected links of a chain. The first step towards enhancing interdisciplinary education is its enculturation, enhancing the spirit of teamwork, and fighting against clichs of superiority of one discipline over the other one [16] or even different levels of a specialty, from general medicine to subspecialty, over other levels. Doctors mostly view themselves superior to their colleagues in terms of intelligence, practical skills, self-reliance, and precision [16]. In fact, the norms must be changed, and the paradigm must shift from individualism to a teamwork attitude [21]. For this to happen, situations for teamwork must be predicted in which students and medical teachers can view the effects of teamwork and gradually incorporate teamwork into their own culture. In morning reports or when encountered with complex scenarios, opportunities can be provided for the exchange of ideas with clinical teachers of different disciplines, so that students would acquire a general view of treatment [22], become familiar with the roles and skills of other professions [23], and realize that success in patient care requires interventions from a wide spectrum of disciplines [24]. In interdisciplinary situations, the spirit of teamwork and cooperation will be strengthened when students receive feedback on their performance and role [24]. This attitude leads to respect for professional roles, enhances cultural sensitivities [25], boosts flexibility, shatters the belief in the superiority of doctors over others, reduces tensions among healthcare professions [26, 27], promotes the professional socialization of students, achieves a common value system, rebuilds and internalizes common interdisciplinary values, and forms an interdisciplinary identity to complement the students professional identity for having an effective, ethical, and safe performance in clinical settings [28], finally promoting teamwork and overcoming the challenges of the healthcare system.

Curriculum developers can equally define values and practical interdisciplinary knowledge in the curriculum for the students of all disciplines, thus helping them to acquire a common value, language, and perception [24]. With such a view towards health sciences, a framework can be created for facilitating communication and teamwork [3]. Several studies have enumerated the main barriers in the implementation of interdisciplinary education as follows: a change in the educational curriculum, identification of settings and situations in the curriculum for involving students of different disciplines in common activities [23, 29], difference in the number of students in diverse disciplines (e.g. large number of medical students compared to other disciplines which leads to dominance of one discipline over the others), lack of compatibility between the levels of education, preliminary capabilities, and academic progress, coordinating the programs, and allocating time and space [30]. One can conclude that performing interdisciplinary activities requires active participation of all healthcare professions.

As a result of the dominant spirit of individualism of some societies, all medical science disciplines mostly take heed of their profession. Based on the literature, in these societies, doctors have less flexibility than those from other disciplines. For example, they see no reason why nurses should be involved in clinical decision making, and feel a sense of superiority to others. They feel that they do not learn anything from other healthcare team members; however, they learn more while interacting with other doctors or medical students [15].

Most clinical teachers in clinical education settings are dissatisfied with presence of students from different educational levels, and their inability to respond to educational needs of all these students in educational rounds is matter of discussion [31]. They assert that in teaching hospitals, the focus is mostly on specialized education, and they regard this setting to be inappropriate for undergraduate medical education. This is because the educational setting consists of specialized and subspecialized hospitals, and medical scenarios include complex issues that doctors do not want to miss [32]. Thus, they discuss complicated issues, thereby ignoring undergraduate medical education. Another issue is that in most teaching hospitals, residents and fellows teach medical students without first acquiring the capabilities and qualifications for teaching, thereby failing to train junior students with capabilities expected of a general practitioner [32]. On the other hand, the students are in a culture that is inclined towards specialization and teaches them to have a good professional status a physician must be specialized [33]. The possibility to harming patients was another category that emerged from the data. A specialty-oriented education eventually harms the patients and increases the costs imposed on them. Through interdisciplinary education, students of medicine and other healthcare professions focus on providing the best treatment for patients, have an open perspective towards different ideas, and pay attention to patient safety [34]. According to the WHO, the most important benefits of interdisciplinary education are increasing patient safety, reducing mortality rate, decreasing tensions among healthcare professions, creating teamwork opportunities, sharing decisions among healthcare workers for achieving general goals, and decision-making about patient management [35]. Studies have also shown that interdisciplinary education is a step forward to achieving a common value, language, and perception, establishing empathy and cooperation among disciplines, and understanding the professional responsibilities of other disciplines. In this regard, this study explored the general viewpoint of medical faculty members and medical students about barriers of multi-professional education in the Iranian teaching hospitals. Thus, it is recommended that the future studies specifically explore views and experiences of faculty members in different teams of specialties and health professions. Also, further studies are required to explore and improve performance of health care teams via multi-professional programs that focus on developing explicit strategies to enhance capabilities of team members and strengthen team-work spirit in Iranian health care contexts.

## Conclusion

Based on the lived experiences of medical faculty members, enculturating a teamwork perspective in healthcare is the first step towards enhancing multi-professional education. It seems that education and healthcare in Iran are on the brink of specialization. As long as cultures do not change, a sudden change in the medical curriculum to a multi-professional approach would be impossible. Enculturation can begin at the micro-level, such as holding morning report sessions, journal clubs, and common sessions between clinical departments. At the macro level, infrastructures for implementation of multi-professional education must also be provided. This requires policy-making by curriculum developers for the development of multi-professional contents, inclusion of multi-professional settings in the curriculum, and designing courses for training clinical teachers with capabilities required for multi-professional education.

## Supplementary Information

**Additional file 1.** Interview Guide.

## Data Availability

Data are available from the authors upon reasonable request and upon the agreement of Iran University of Medical Sciences vice-deputy for Research.

## Abbreviations

EDC: Educational Development Center
WHO: World Health Organization
MOHME: Ministry of Health and Medical Education
