# Supplementary material for: A qualitative approach to identify barriers to multi-professional teamwork among medical professors at Iranian teaching hospitals
Source: BMC Health Serv Res. 2021 May 20;21:479. doi: 10.1186/s12913-021-06421-4 (PMC8139062; doi:10.1186/s12913-021-06421-4)
Supplement: Supplementary file 1 — Additional file 1. Interview Guide. [file 12913_2021_6421_MOESM1_ESM.docx]

**Interview Guide**

"Good morning. I am Hakimeh Hazrati.

This interview is being conducted to get your experiences of multi-professional teamwork barriers among medical professors at Iranian teaching hospitals. The project is part of PhD thesis entitled “Designing A Customized Model of Effective Clinical Teaching for an Undergraduate Medical Program: A Grounded Theory Study” with ethics code of) IR.IUMS.FMD.REC.1398.217 (registered by Research Ethics Committee of Iran University.

"If it is okay with you, I will be tape recording our conversation. The purpose of this study is so that I can get all the details but at the same time be able to carry on an attentive conversation with you. I assure you that all your comments will remain confidential. If you agree to this interview and the tape recording, please sign this consent form."

"I'm now going to ask you some questions that I would like you to answer to the best of your ability.”

1. “Can you talk about one of your best multi-disciplinary education experiences, in an outpatient setting or at the bedside in in-patient settings, for medical students?

Probing questions:

- 1. “What were the characteristic*s of this experience?"*
  2. *Could you please explain more?*

1. "Are you aware of any multi - disciplinary teaching problems in Iranian teaching hospitals?

Probing questions:

- 1. "Do you know why these problems are occurring?"
  2. "Do you have any suggestions on how to minimize these problems?"

1. "How can we implement multi - disciplinary education in Iranian teaching hospitals/settings?"
